# Profile of risk factors associated with suicide attempts: A study from Orissa, India

**DOI:** 10.4103/0019-5545.58895

**Published:** 2010

**Authors:** Nilamadhab Kar

**Affiliations:** Mental Health Directorate, Wolverhampton City Primary Care Trust, Corner House Resource Centre, 300, Dunstall Road, Wolverhampton, WV6 0NZ, UK

**Keywords:** Attempted suicide, developing countries, risk factors

## Abstract

**Context:**

Periodic systematic profiling of suicidal risk factors in developing countries is an established need.

**Aims:**

It was intended to study the risk factors associated with suicide attempts in Orissa, one of the most economically compromised states of India.

**Settings and Design:**

Cross-sectional study in a general hospital.

**Materials and Methods:**

Consecutive 149 suicide attempters were evaluated for psychosocial, situational, and clinical risk factors using the Risk Rescue Rating scale, Suicide Prevention Center scale, Lethality of Suicide Rating scale, and Presumptive Stressful Life Event scale. They were compared with healthy and psychiatric controls who had never attempted suicide.

**Statistical analysis:**

Chi-square for comparison of categorical variables, *t*-tests for comparison of means.

**Results:**

The male-to-female ratio was closer to one in adults and around 1:3 in adolescents. Younger age, lower-middle economic group, rural background, unemployed, school educated were more represented in this study. Compared to the controls, significantly more number of attempters had a family history of psychiatric illness and suicide, childhood trauma, medical consultation within one month, had experienced stressful life events and had expressed suicidal ideas. In a considerable proportion of attempts, risk was high and rescuability least; 59.1% had more than 50% chance of death. Suicide potential was high in almost half the cases. More than 80% of all attempters had psychiatric disorder; however, only 31.5% had had treatment.

Factors like middle age, family history of psychiatric disorders, past psychiatric history, current psychiatric illness, communication of suicidal ideas, the use of physical methods, and high potential attempts, differentiated repeaters significantly from the first-timers. Major physical illness, family and marital conflicts, financial problems, and failure in examinations were more frequent life events. Childhood trauma, noted in around 40% of the attempters, was considerably associated with adolescent suicide attempts.

**Conclusions:**

Modifiable risk factors identified in this study have preventive implications.

## INTRODUCTION

The true magnitude of suicide as a public health problem is not clear in India. In the last two decades, official figures of suicide rate in India have increased from 7.9 to 10.3 per 100,000.[1] The actual number of suicides is understandably more than the reported official figures as non-reporting, under-reporting, and misclassification are prevalent due to various socio-cultural stigmas, religious sanctions, legal issues, and insufficient registration systems.[2] It has been suggested that the annual suicide rate could be six to nine times the official rate.[1] There has been less work in systematic profiling of risk factors in developing countries compared to the developed counterparts.[3] Variations in suicide risk factors in different cultures and periods are known;[4,5] and it is acknowledged that more research is required, especially from developing nations.[3,6]

Orissa, one of the poorest states in the East coast of India, has a reported suicide rate of 10.3 to 10.4 per 100,000 in the last decade.[7] The percentage of rural families living below the poverty line (66.4%) is much higher in the state. The state faces economic hardship due to floods, cyclones, droughts, and has destitute poverty of persons lacking either money or material to survive. Developmental indicators and the living conditions of the people of the state are considerably lower than the national average.[8] In the above context, it was intended to study the risk factors associated with suicide attempts in a sample from Orissa and to reflect on the possible prevention methods. The specific objectives of this study were to evaluate socio-demographic variables, clinical and situational factors, as well as the methods and severity of attempts.

## MATERIALS AND METHODS

The study was carried out in the SCB Medical College Hospital, Cuttack. ‘Any act of self damage inflicted with self destructive intentions, however vague and ambiguous’ was taken as a suicide attempt,[9] for the purpose of the study. Consecutive 149 suicide attempters referred from medical and surgical departments and casualty over a period of two years (July 1994 to June 1996) were taken up for the study. All the suicide attempters admitted to the hospital were referred for psychiatric evaluation considering medico-legal issues. In addition, the departments were informed about the study to ensure prompt referral. Patients whose injuries were considered to be accidental in origin with no suggestion of self harm intention, and those succumbed to their injuries, were excluded from the study. The patients were interviewed once they gained physical stability after resuscitation and a period of observation in the medical or surgical unit. Close family members of each patient were interviewed with the patients' consent for additional information.

Two control groups comprising of psychiatric patients (*n* = 40) and healthy individuals (*n* = 45) were selected on the criterion of never having attempted suicide. With the help of a Table for random numbers, two separate lists for each control were generated from the registration numbers of patients attending the Department of Psychiatry. The study was explained to selected individuals, and those who fulfilled the criterion and agreed to participate, were included. Healthy controls were selected from the friends (not relatives) accompanying the patients.

The study protocol was approved by the Institutional Ethics Committee. Informed consent was collected from the participants and confidentiality was assured. A semi-structured proforma was used for recording the socio-demographic profile, methods and situations around the suicide attempt, intent, communication method, and clinical profile of the patient. Medical consultations one month before the attempt, physical illness, family history of psychiatric illness and suicide, past psychiatric history, addiction, and past psychiatric treatment were specifically enquired for. Childhood trauma characterized by the loss of a parent before the age of 15, perceived lack of adequate parental care, alcoholism in a parent, parental conflict, and sexual abuse were noted. Psychiatric diagnoses were made according to diagnostic criteria for research of ICD-10 Classification of Mental and Behavioral Disorders.[10]

Suicide potential was assessed by the Los Angeles Suicide Prevention Center Scale[11] which is designed to aid in the evaluation of risk factors. The score suggests a general level of self-destructive status which is categorized as low, medium, or high potential. Lethality of the suicide attempt, *e*.*g*., ‘the possibility or degree to which any biological change that could have endangered the life of the patients if not rescued and resuscitated’ was evaluated by the Lethality of Suicide Attempt Rating scale.[12] It is a 11-point scale starting from 0.0, *i*.*e*., death is an impossible result of the suicidal behaviour to 10.0, *i*.*e*., death is almost a certainty. It is a composite of the actual lethality of the method used and the circumstances surrounding the attempt.

Risk and rescue factors associated with the suicide attempt were studied by the Risk and Rescue rating scale.[13] Risk factors included were the agents used, level of consciousness, lesions, toxicity, reversibility, and treatment required. Rescue factors studied were locations, person initiating rescue, and probability of discovery, accessibility for rescue, and delay until discovery. They were rated on a three-point scale and referred in five grades of risk or rescue. Life events (LEs) within one, six, and 12 months before the attempt were studied using the Presumptive Stressful Life Event scale (PSLES)[14] which provides stress scores for different events.

The associations among categorical variables were analyzed by chi-square tests; Yate's correction was done when necessary. The differences in the means were compared by *t*-tests and the threshold of statistical significance was at standard 0.05.

## RESULTS

The socio-demographic profile of attempters is given in Table 1. The comparison of clinical characteristics with the healthy and psychiatric controls is presented in Table 2. There was no refusal for participation in the study by the attempters, their family, or the controls. Gender distribution of the attempters and the control groups (healthy: 12 males, 33 females; and psychiatric patients: 14 males, 26 females) was comparable. The mean age of the attempters (31.6 ± 13.5 years) was not significantly different to that of healthy (33.2 ± 10.1) and psychiatric (29.5 ± 9.8) controls. Information regarding the suicide attempt is given in Table 3. Pesticides (44.3%), oleander (31.3%), and medicines (24.5%) were more frequently used substances for poisoning.

**Table 1:** Socio-demographic characteristics

Variables	Male (*n* = 65)		Female (*n* = 84)		Total (*n* = 149)	
			
	*n*	%	*n*	%	*n*	%
Age (years)*							
≤ 19	5	7.6	17	20.2	22	14.7
20-29	23	35.3	32	38.1	55	36.9
30-39	21	32.3	19	22.6	40	26.8
40-49	6	9.2	4	4.7	10	6.7
50-59	7	10.7	6	7.1	13	8.7
≥ 60	3	4.6	6	7.1	9	6.0
Religion						
Hindu	63	96.9	84	100.0	147	98.6
Marital status						
Unmarried	27	41.5	26	30.9	53	35.5
Married	37	56.9	51	60.7	88	59.1
Widowed/Separated	1	1.5	7	8.2	8	5.3
Habitat						
Rural	51	78.4	57	67.8	108	72.4
Occupation						
Student	13	20.0	17	20.2	30	20.1
Housewife	-	-	53	63.1	53	35.5
Unemployed†	9	13.8	11	13.1	20	13.4
Laborer/Skilled worker	29	44.6	1	1.2	30	20.1
Office/business	14	21.4	2	2.3	16	10.7
Economic status						
Lower	3	4.6	3	3.5	6	4.0
Middle	60	92.2	76	90.4	136	91.2
Upper	2	3.1	5	5.9	7	4.7
Education level (years)						
No formal education (0)	0	0.0	12	14.2	12	8.0
School (1-9)	34	52.3	44	52.4	78	52.3
College (10-14)	28	43.1	25	29.7	53	35.5
University (15)	3	4.6	3	3.5	6	4.0
Family						
Extended	59	90.7	71	84.5	130	87.2
Nuclear/Living alone	6	6.2	13	15.4	19	13.0

* Range: 12-70 years;

† Employed *vs* non-employed, *P* < 0.001

**Table 2:** Comparison with controls

Variables	Suicide attempters (*n* = 149)		Healthy control (*n* = 45)		Psychiatric control (*n* = 40)	
			
	*n*	%	*n*	%	*n*	%
FH of psychiatric illness*	83	55.6	4	8.8	36	90.0
FH of suicide	20	13.3	1	2.2	3	7.5
PH psychiatric illness	47	31.5	0	0.0	12	30.0
Childhood trauma*	59	39.6	4	8.8	4	10.0
Medical consultation within one month*	60	40.2	11	24.4	2	5.0
Communicated idea*	110	73.8	6	13.3	13	32.5
Number of persons reporting LEs						
One month*	131	87.9	9	20.0	4	10.0
Six months*	146	96.9	19	42.2	17	42.5
One year*	147	98.6	27	60.0	32	80.0
Mean LE score (SD)						
One month*	62.5	(24.8)	53.3	(1.8)	38.0	(9.0)
Six months*	97.2	(47.5)	51.0	(10.9)	46.1	(9.3)
One year*	118.8	(60.3)	55.8	(20.6)	58.0	(18.4)

FH - Family history; PH - Past history; LE - Life events, SD - Standard deviation

* *P* < 0.001

**Table 3:** Characteristics of suicide attempt

Variables	Male		Female		Total	
			
	*n*	%	*n*	%	*n*	%
Method						
Poisoning	22	33.8	39	46.4	61	40.9
Hanging	29	44.6	23	27.3	52	34.9
Drowning	3	4.6	11	13.1	14	9.4
Railway/road	4	6.1	2	2.3	6	4.0
Stabbing/Slashing	1	1.5	4	4.6	5	3.3
Setting fire	1	1.5	3	3.5	4	2.6
Firing	3	4.6	0	0.0	3	2.0
Electrocution	1	1.5	1	1.2	2	1.3
Jumping	1	1.5	1	1.2	2	1.3
Easy availability of method	62	95.3	83	98.8	145	97.3
Alcohol before attempt	7	10.7	5	5.9	12	8.0
Planned attempt*	44	67.6	33	39.2	77	51.6
Unequivocal intent to die	25	38.4	29	34.5	54	36.2
Communication†						
Ideation	48	73.8	62	73.8	110	73.8
Threat	6	9.2	10	11.9	16	10.7
Notes	8	12.3	8	9.5	16	10.7
Lethality score‡						
< 5	8	12.3	9	10.7	17	11.4
5	18	27.6	26	30.9	44	29.5
>5	39	60.0	49	58.3	88	59.1
Risk-rating score						
High risk	21	32.3	28	33.3	49	32.8
High moderate	16	24.6	21	25.0	37	24.8
Moderate	9	13.8	19	22.6	28	18.7
Low moderate	17	26.1	9	10.7	26	17.4
Low risk	2	3.1	7	8.3	9	6.0
Rescue-rating score					
Least rescuable	16	24.6	21	25.0	37	24.8
Low moderate	14	21.5	21	25.0	35	23.4
Moderate	24	36.9	27	32.1	51	34.2
High moderate	9	13.8	14	16.6	23	15.4
Most rescuable	2	3.1	1	1.2	3	2.0
Suicide potential§						
Medium	27	41.5	52	61.9	79	53.0
High	38	58.4	32	38.1	70	46.9

* *P* < 0.001;

† no communication in 7 attempters;

‡ Lethality score: 5 - death probability fifty-fifty;

§ *P* < 0.02

### Socio-demographic variables

Family and past history of psychiatric illness and attempt, childhood trauma, addiction, past attempts, current psychiatric and physical illness, nature of attempt, communication of suicidal intent, potentiality, lethality, risk and rescue factors, method of attempt, and LE scores were compared in various socio-demographic groups. Variables in which the groups differed significantly are described.

#### Age and gender

The age group of 20-29 years was most commonly represented in both genders. In adult attempters, the male-female ratio was 1: 1.1 whereas it was 1: 3.4 in adolescents. Attempters were categorized as adolescents, young adults, middle-aged, and older adults for comparison. Variables in which the age groups differed significantly are presented in Table 4.

**Table 4:** Clinical characteristics of different age groups of attempters

Variables	<19 (*n* = 22)		20-39 (*n* = 95)		40-64 (*n* = 23)		65< (*n* = 9)	
				
	*n*	%	*n*	%	*n*	%	*n*	%
Childhood trauma*	14	63.6	31	32.6	11	47.8	3	33.3
PH psychiatric illness†	3	13.6	25	26.3	16	69.6	3	33.3
PH of suicide attempt‡	5	22.7	53	55.8	18	78.3	4	44.4
Current psychiatric illness†	8	36.4	83	87.4	23	100.0	9	100.0
Current major physical illness*	3	13.6	21	22.1	10	43.5	5	55.5
Impulsive attempt†	20	90.9	44	46.3	2	8.7	6	66.6
Expressed suicidal idea‡	10	45.5	75	78.9	20	86.9	5	55.5
High potential†	2	9.1	47	49.5	17	73.9	4	44.4

* *P* < 0.05

† *P* < 0.001;

‡ *P* < 0.01

PH - Past history (only significant findings)

#### Habitat

Most of the attempters were from rural areas. Significantly more attempters from urban backgrounds reported childhood trauma (53.6 *vs* 34.2%, *P* < 0.05), had addiction (24.3 *vs* 6.4%, *P* < 0.01), wrote suicide notes (19.5 *vs* 7.4%, *P* < 0.05) and took alcohol before the attempt (17.0 *vs* 4.6%, *P* < 0.02) compared to those from rural areas. However, a significantly higher proportion of rural attempts (30.5 *vs* 9.7%, *P* < 0.01) were least rescuable.

#### Educational status

There was no significant difference in the educational groups regarding the variables studied, except that persons with no formal or with college education had around 40% attempts that were least rescuable, which was significantly more than that from the other educational groups (*P* < 0.01).

#### Employment

A sizable proportion of the attempters (30.3%, excluding students and housewives) were unemployed. A comparison of employed (*n* = 46) and unemployed (*n* = 20) attempters revealed that significantly more of the employed subjects had planned attempts (76.1 *vs* 45.0% respectively, *P* < 0.02) and had higher stress scores (*P* < 0.02) within one month before their suicide attempts.

#### Marital status

Psychiatric past history (39.7%) and addiction (15.9%) were significantly (*P* < 0.05) more common in the married group. Attempts of most (75.0%) of the widowed persons and 53.4% married persons were of high potential compared to that (32.0%) of the unmarried (*P* < 0.02). The mean stress scores of married attempters were more than that of the unmarried one month before the attempt (*P* < 0.001), whereas the mean stress scores of widowed persons were more in the 12 month period before the attempt (*P* < 0.05). All the widowed attempters had mental illness, compared to 88.6% of the married and 69.8% of the unmarried (*P* < 0.01).

#### Family type

The proportion of attempters from extended families with psychiatric illness (86.1%) was more than that (52.9%) from the nuclear families (*P* < 0.001), and most of them had expressed suicidal ideas (76.9 *vs* 47.0% from the nuclear) (*P* < 0.01). Stress scores of attempters from extended families were more within one month prior to the attempt (*P* < 0.001).

#### Socioeconomic status

Most of the attempters had a middle socioeconomic status (SES), amongst which the majority (86.8%) were lower-middle. Significantly more attempters with upper SES reported psychiatric illness (*P* < 0.05), physical illness (*P* < 0.05), childhood trauma (*P* < 0.05), took alcohol before the attempt (*P* < 0.001), and had significantly high stress scores one and 12 months prior to the attempt (*P* < 0.05). Stress scores of LE were minimum in the lower SES in all the three time periods studied.

### Clinical variables

Clinical characteristics of the sample, including the reports of childhood trauma, are given in Table 5. System-wise break-up of major physical illness included: Gastrointestinal (7.3%), urogenital and respiratory (3.3% each), central nervous system, musculoskeletal and endocrine (2.6% each), cardiovascular and organic sexual problems (2.0% each). Significantly more number of attempters with major physical illness reported family history of suicide (17.9 *vs* 6.3%) compared to those with no physical illness (*P* < 0.05). Current principal psychiatric diagnoses of the attempters have been given in Table 6.

**Table 5:** Clinical characteristics of the sample

Variables	Male		Female		Total	
			
	*n*	%	*n*	%	*n*	%
FH of psychiatric illness	28	42.6	55	55.4	83	55.6
FH of suicide	9	13.8	11	13.0	20	13.3
PH psychiatric illness	25	38.4	22	36.1	47	31.5
PH of psychiatric treatment	25	38.4	22	26.1	47	31.5
PH of suicide attempt	38	58.4	42	50.0	80	53.6
Current psychiatric illness	57	87.6	66	78.5	123	82.5
Current substance use*	14	21.5	3	3.5	17	11.4
Medical consultation within one month	25	38.4	35	41.6	60	40.2
Current major physical illness	15	23.1	24	28.5	39	26.1
Childhood trauma						
Not mentioned	37	56.9	53	63.1	90	60.4
Lack of adequate parental care	13	20.0	9	10.7	22	14.7
Loss of mother before 15	8	12.3	7	8.3	15	10.1
Loss of father before 15	4	6.1	7	8.3	11	7.3
Alcoholism in parent	1	1.5	6	7.1	7	4.7
Parental conflict	2	3.1	0	0.0	2	1.3
Sexual abuse	0	0.0	2	2.3	2	1.3

FH - Family history; PH - Past history;

* *P* < 0.001

**Table 6:** Psychiatric diagnoses

Variables	Male		Female		Total	
			
	*n*	%	*n*	%	*n*	%
No diagnosis	8	12.3	18	21.4	26	17.4
Substance abuse/dependence	5	7.6	0	0.0	5	3.4
Schizophrenia	8	12.3	11	13.1	19	12.7
Acute and transient psychotic disorder	2	3.1	8	9.5	10	6.7
Psychosis other and unspecified	13	20.0	8	9.5	21	14.1
Bipolar mania	2	3.1	1	1.2	3	2.0
Bipolar depression	2	3.1	1	1.2	3	2.0
Depressive disorders	15	23.1	22	26.2	37	24.8
Obsessive compulsive disorder	1	1.5	1	1.2	2	2.0
Adjustment disorder	9	13.8	11	13.1	20	13.4
Somatisation disorder	0	0.0	1	1.2	1	0.6
Personality disorder	0	0.0	2	2.4	2	2.0

#### Mental illness

Compared to those with no psychiatric disorders, attempters with psychiatric disorder had significantly more past psychiatric history (3.8 *vs* 37.4%, *P* < 0.001), prior attempts (11.5 *vs* 62.6%, *P* < 0.001), planned attempts (23.1 vs 57.8%, *P* < 0.01), expression of suicidal ideas (19.2 vs 85.3%, *P* < 0.001), high potential attempts (26.9 *vs* 51.2%, *P* < 0.05), used physical methods (26.9 *vs* 65.8%, *P* < 0.001), and had high stress scores one month prior to the attempt (*P* < 0.01). In contrast, the proportion of high risk attempts was more in those without psychiatric disorder (50.0 *vs* 29.2%, *P* < 0.05).

Attempters with mood disorder (n = 43) had more planned attempts (83.8 *vs* 43.8%) compared to those with other psychiatric disorders (n = 80). The former also had significanstly more high potential attempts (67.4 *vs* 42.5%, *P* < 0.01) and used more chemical methods (53.4 *vs* 23.7%, *P* < 0.001). The latter had significantly more stress scores one month prior to the attempt (*P* < 0.01).

Compared to attempters without substance abuse, those with it (n = 17: Five as primary and 12 as comorbid) reported the following more frequently: Family history of suicide (6.8 *vs* 29.4%, *P* < 0.01), prior attempt (50.7 *vs* 76.4%, *P* < 0.05), planned attempt (48.5 *vs* 76.5%, *P* < 0.05), left suicide notes (6.8 *vs* 41.1%, *P* < 0.001), took alcohol before the attempt (5.3 *vs* 29.4%, *P* < 0.001), high potential attempts (43.9 *vs* 70.5%, *P* < 0.05), used physical methods (57.5 *vs* 70.5%, *P* < 0.05) and had higher stress scores one month prior to the attempt (*P* < 0.05). Alcohol (80%) and cannabis (20%) were the substances involved.

#### Previous suicide attempt

Comparison between attempters with a past history of suicide attempt(s), and those without any such past history, revealed more frequently that repeaters had past psychiatric history (40.0 *vs* 21.7%, *P* < 0.02), addiction (16.2 *vs* 5.8%, *P* < 0.05), psychiatric illness (96.2 *vs* 66.6%, *P* < 0.001), planned attempt (63.8 *vs* 37.7%, *P* < 0.01), expression of suicidal ideas (88.7 *vs* 56.5%, *P* < 0.001), high potential attempts (57.5 *vs* 34.7%, *P* < 0.01), use of physical methods (73.7 *vs* 42.0%, *P* < 0.001) and more stress scores one month prior to the attempt (*P* < 0.01).

#### Intent to die

Compared to those who were ambivalent, attempters with unequivocal intent to die had proportionately more psychiatric illness (77.8 *vs* 90.7%, *P* < 0.05), planned attempt (39.0 *vs* 74.1%, *P* < 0.001), higher (score more than 5) lethality (44.2 *vs* 85.1%, *P* < 0.001), high risk (23.1 *vs* 50.0%, *P* < 0.001) and higher stress scores in all three time periods studied.

#### Stressful life events

The nature of stressful life events experienced by the attempters is given in Table 7. Comparison of the mean scores of LE with those of the controls during one, six, and 12 months prior to the attempt has been given in Table 2.

**Table 7:** Nature of life events*

Variables	Male		Female		Total	
			
	*n*	%	*n*	%	*n*	%
Major personal illness	30	46.2	35	41.6	65	43.6
Family conflict	20	30.7	35	41.6	55	36.9
Marriage-related LE	13	20.0	39	46.4	52	34.9
Financial loss/problem	15	23.1	8	9.5	23	15.4
Minor violation of law	9	13.8	12	14.2	21	14.1
Failure in examination	5	7.6	10	11.9	15	10.1
Death of family member	5	7.6	7	8.3	12	8.1
Work-related LE	9	13.8	2	2.3	11	7.4
Unemployment: Self/family member	7	10.7	2	2.3	9	6.0
Sexual problems	3	4.6	4	4.7	7	4.7

* more than 4% in the sample; LE: Life events

## DISCUSSION

Risk factors observed to be associated with the suicide attempts in this study were consistent with those reported commonly in literature. However, there were variations and the results suggested locally relevant issues that can contribute to prevention strategies.

### Socio-demographic risk factors

#### Age

A preponderance of younger age groups is frequently noted in suicide attempts.[15,16] In this index study' around half of the attempters were below 30 years of age and more than 75% were below 40. All the attempters aged more than 40 years had been diagnosed with psychiatric disorders. Prior and planned attempts underscored the risk of middle-aged adults. Adolescents were distinct from other age groups as 90% of them attempted impulsively with only 9% of the attempts being of high potential. Poor impulse control has been reported in adolescent suicidal behaviours.[17] The proportion of adolescents with mental illness was the least whereas the report of childhood trauma was most frequent in adolescents; and all of them reported LEs. It is known that LEs play a major role in adolescent suicide attempts.[18] The LEs were mostly failure in examinations and minor violations of discipline with anticipation of negative repercussions. Failure in examinations has been associated with suicidal behaviour.[19] As the outcome of examinations virtually decides an individual's future, failures become extremely stressful. It appears that attempts in adults were mostly secondary to psychiatric illness and were serious, in contrast to those of adolescents and young adults which were mostly impulsive and secondary to anomalous life conditions.

#### Gender

The male-to-female ratio was closer to one in adults in this study. There are reports of both male and female predominance in suicide attempts in hospital-based studies.[4,20] The gap between male and female suicide rates in India is relatively small.[21] In adolescents, however, the female proportion was more than 75%; similar findings have been reported in India and elsewhere.[16,18] Females were over-represented below 30 years of age after which there was male preponderance. This reversal of pattern has been observed in the West for single men and women.[16] The genders did not differ regarding lethality, or risk and rescue factors; except that attempts by males were of high potential probably because more males planned in contrast to most females whose attempts were impulsive. Lethality of the attempts by both genders was comparable in another Indian study.[22]

#### Habitat

Rural-urban differences in SES, access to means, as well as access to and facilities of health services may all contribute to varying rates of suicide that have been reported from Indian studies.[22,23] Most of the attempters in this study were from rural areas. Although there was no significant difference in the methods used or the lethality, the attempts in rural areas were least rescuable compared to those in urban areas. Urban attempters were distinct in more frequently reporting childhood trauma, addiction, use of alcohol before the attempt, and writing suicide notes.

#### Employment status

A considerable proportion of attempters in this study were unemployed. Unemployment is a known risk factor for suicide attempts.[15,24,25] In the highly competitive situation for jobs in India, being unemployed is extremely stressful. However, employed attempters had significantly higher stress scores than the unemployed, which might explain partly their reason for attempt. Considering the type of employment, skilled workers were most represented, followed by the self-employed and professionals. These work groups probably experience job uncertainties more often than those in salaried government jobs.

#### Socioeconomic status

In this study, the lower-middle SES was the most predominant in the attempters. Lower economic strata[25,26] and poverty have been associated with suicide attempts.[3] In the fast-changing economic scenario, those in the lower-middle SES are highly stressed, which probably makes them the most vulnerable to suicide attempts.

#### Marital status

Being single, divorced or separated, or widowed have been found to be risk factors in many Western studies[16] although it may not be predictive in developing countries.[3] A small minority (around 5%) were separated or widowed and a sizeable proportion was unmarried. Not being able to get married is a significant stress, especially for females and their families in India, which has been found to be associated with suicide.[27] However, in Indian studies, it is common to find a higher proportion of attempters being married, as observed in this study.[15] A considerable proportion of attempters had LEs related to relationships and marriage, *e*.*g*., broken love affairs, getting married, conflict with in-laws, marital conflict, separation, divorce, and extramarital relationship of the spouse. Marital/relational problems have been frequently reported in Indian studies,[1,27,28,29,30] as else where,[16,31,32] in connection with suicide attempts.

#### Family type

Varieties of family patterns have been described that predispose an individual to suicidal behaviour.[33] Attempts by persons from extended families were mostly attributed to mental illness and stress in contrast to those from nuclear families. This suggests that factors specific to nuclear families might be involved in many attempts, which would need further study. As the traditional joint family system in India is functionally changing to nuclear units, the risk might be changing too.

### Environmental/situational risk factors

#### Methods

Poisoning, hanging, and drowning, as observed in this study, have been the common methods for suicide attempts in India.[15,25,20] Poisoning was used more frequently by adolescents, first-time attempters, depressives, and attempters without psychiatric disorders. Physical methods were more frequently used by those with unequivocal intent to die, those from extended families, repeaters, substance abusers, and psychiatric patients other than depressives.

#### Access to methods

Access to methods of suicide was reported to be easy by most of the attempters; which precludes prevention by restricting the methods. Restriction of access to the methods of suicide has received some attention as a possible way of prevention of suicidal death.[34] However, it has been observed in an Indian study that when the use of pesticide was restricted, the mode of suicide changed while the total number of suicides remained static.[28] Nevertheless, as poisoning was the most common method of suicide attempt; and pesticides were used most frequently; restricting the availability of organophosphorus compounds, banning the more toxic ones, as well as efforts to decrease the period between the ingestion and initiation of treatment by having poisoning treatment facilities in primary health care centers may be helpful in preventing or lowering the rate of suicidal attempts.[35]

#### Life events

Reports of LEs and their severity in attempters were significantly more than in the healthy and psychiatric controls within one year prior to the attempts. This underscores the role of higher density and severity of LE in suicide attempts, as in other studies.[25,36] Major physical ailments were the most frequently reported stressors, known to be associated with suicide attempts.[27] It may be highlighted that medical treatment is a financially burdensome affair for the majority of people in this developing state. As found in other studies, financial problems were noted as a major stress in this study.[25,16,29,32] Financial setbacks are well reported as a major cause of suicide in Indian farmers.[37] Most of the attempters in the economically compromised state of Orissa were in the lower-middle SES, which is another cause for concern. Other notable LEs were related to marriage/relationships, work, unemployment, and death of spouse or family members; which are commonly reported to be associated with suicidality.[7,15,20,25]

#### Childhood trauma

Childhood trauma has been shown to be significantly associated with suicidal behaviour.[38] Lack of parents, perceived inadequacy in care, and alcoholism in a parent were more common themes. Negative parental rearing, parental loss,[25,39,40] negative perception of parents;[40] and physical abuse have been reported as factors for adolescent suicide attempts. However, there were no parental divorce/separation instances in this study as they are uncommon in Orissa. Childhood sexual abuse which has been reported to be associated with suicidal behavior,[41] was rarely reported in this study.

### Clinical risk factors

#### Mental illness

A majority (82.5%) of the attempters in this study had psychiatric illness. Although hospital studies in India have reported similar figures;[20,25,42] this is higher than the figures reported in the West.[16] A variation in the type and frequency of the psychiatric disorders is noted in suicide attempters in India, although depressive disorders are common.[20,42,43,44] In this study, mood disorders were common, with depressive disorders being most prominent. One in three had a psychotic disorder, which is higher than usually reported figures.[16] Personality disorder was hardly diagnosed in this study; however, it may need focused assessment. Only 31.5% of the sample had psychiatric intervention which is slightly less than the reported figures of around 40% elsewhere.[16] Patients with mood disorder were more vulnerable than others considering planned attempts of high potential, even though most of them used chemical methods. Attempts of patients with non-mood disorders were associated with significantly higher stress scores one month prior to the attempts, suggesting a contributing role of LEs.

Substance abuse was less frequently reported in this study than elsewhere.[25,29,36] However, attempts by the persons with substance abuse were significantly associated with several risk factors like family history of suicide, childhood trauma, prior attempts, planned attempts, use of physical methods, and taking alcohol before the attempts, suggesting a higher vulnerability of this group. Association of substance abuse and suicidality is well known.[25,36,45]

#### Physical illness

Characteristics of most of the associated physical illnesses were chronic, nonremitting pain, restriction of occupational and recreational endeavors, and physical mobility. Persons with major physical illness and family history of suicide were probably particularly vulnerable considering the observed significant association. A considerable proportion (40%) of attempters sought medical consultation one month before the attempt; the frequencies elsewhere vary from 25 to 80%.[46] About one third (31.6%) of those who went for consultation had psychiatric disorders. These findings suggest the probability of some scope for early identification of suicide risk in this population.

#### Suicidal communication

A considerable proportion of attempters communicated their intentions. The suicidal ideation was associated significantly with adult attempters, psychiatric patients, repeaters, and those from extended families. Expression of ideas did not differentiate the level of intent, but these were found significantly more with attempters compared to the controls. This underscores the significance of suicidal communication as cues that are relevant in the identification of vulnerable persons.

#### Lethality

Lethality of the attempts varied considerably, with the majority (60%) having more than 50% chance of death. Most of the attempts in Indian studies have been found to be of higher lethality[15,18,35,47] with few variations.[4] Besides the physical methods, a majority of organophosphorus poisoning cases were also associated with higher lethality. Lethality was comparable between the genders, as reported in another study.[48] Only a small minority of attempters used alcohol before the attempts, something that is known to influence the risk. Females used it too in other studies, which was not noted in studies from India.[4,49]

#### Intent

A little over one third of the attempters reported unequivocal intent to die; similar to reported frequencies of around 33 to 41%.[22,23,48] Intent to die has been associated with psychiatric illness, higher life event scores, and drastic and instantaneous methods of higher lethality with dangerous medical consequences.[23,32,48] In this study, attempters with unequivocal intent to die had significantly higher lethality and risk than those were ambivalent. There were no differences between the genders regarding suicidal intention, unlike studies that report males having higher intent.[48] It was evident that the ones who were more determined to die, had planned their attempts, and the planned attempt was also significantly associated with persons with psychiatric illness, especially depressives and substance abusers.

#### Risk and rescue factors

A significant proportion of attempts was of high risk and was least rescuable. Risk was greater in the attempters without psychiatric illness and in those with an unequivocal intent to die. In rural areas where the delay until intervention can be considerable, the rescuability was low. High impulsivity and a low level of communication have made the attempts of those without psychiatric disorder carry a high risk.

#### Potentiality

It was found that males, middle aged adults, unmarried persons, those with psychiatric illness, particularly depression, substance abusers, and repeaters had made comparatively more high potential attempts. Vulnerability of these groups is underscored in this study.

#### Repeat attempt

Multiple attempters were reported to have chronic symptoms, poor coping skills, family history of suicidal behaviour, substance abuse, and less impulsiveness.[45] The findings of this study suggested that repeaters constituted a distinct group of middle-aged patients, with high family loading of psychiatric disorders, past psychiatric history, current addiction and psychiatric illness, who expressed suicidal ideas, used physical methods, and had high potential attempts, significantly more frequently than first-timers. However, lethality, risk and rescuability of repeaters and first-timers were comparable. Identifying repeaters is important as their rate and probability of death by suicide are significantly higher.[5,50]

#### Comparison with controls

A higher frequency of family history of psychiatric illness and suicide, childhood trauma, medical consultation within one month, LEs and their severity, and expression of a suicidal idea, differentiated the attempters from the healthy and psychiatric controls. Higher numbers of LEs have been reported in attempters as compared to psychiatric patients, which were significantly more than that in the general population.[16] Differentiation not only highlights the risk factors, but also suggests the plausibility of identifying potential attempters, something that needs further study.

### Limitations

The study findings reflect the profile of suicide attempters attending tertiary level hospitals and may not be generalized to all suicide attempters in the general population. There may be a recall bias for childhood trauma and family and past histories. The sample size of the controls was small. Although none of the patients referred for the study refused to participate, the numbers of attempters who were not referred or who died, were not collected.

## CONCLUSIONS

Psychosocial and clinical risk factors associated with suicide attempts in Orissa resembled those described in literature, but with a few variations. Most of the attempters had psychiatric disorders and only a few had psychiatric consultation. Public education for early identification and help-seeking for mental disorders, awareness regarding this in health care staff, and facilities for management of common mental disorders in rural areas would probably help. Restriction of availability of highly toxic pesticides may decrease the lethality of many attempts. Supportive measures for various stressors and interventions for many modifiable risk factors identified, seem plausible and might be considered as a priority in local suicide prevention strategies. The findings of this exploratory study also identify areas for further focused research.
